# Association of Hypertension with Different Cognitive Disorders

**DOI:** 10.3390/jcm13206029

**Published:** 2024-10-10

**Authors:** Lillian Huang, Wilbert S. Aronow

**Affiliations:** 1 Department of Medicine, New York Medical College, Valhalla, NY 10595, USA; 2 Department of Cardiology, Westchester Medical Center, Valhalla, NY 10595, USA

**Keywords:** hypertension, antihypertensive, dementia, Alzheimer’s, vascular, Lewy body, frontotemporal

## Abstract

This literature review explores the association between hypertension and major neurocognitive disorders, including delirium, Alzheimer’s disease, vascular dementia, Lewy body dementia, and frontotemporal dementia, which contribute significantly to global mortality and morbidity. Hypertension is a potentially modifiable risk factor for cognitive decline, as it contributes to the progression of neurodegenerative pathologies via vascular damage, inflammation, and the disruption of the blood–brain barrier. Despite this, the effectiveness of antihypertensive treatments in preventing or alleviating cognitive decline remains contentious. While some research highlights the potential benefits of angiotensin-converting enzyme inhibitors and angiotensin receptor blockers, other studies show inconsistent results, complicated by variations in hypertension definitions, diagnostic criteria for cognitive disorders, and confounding factors like medication adherence. Furthermore, the complex bidirectional relationship between hypertension and major neurocognitive disorders warrants more investigation, as cognitive decline can exacerbate cardiovascular risks through heightened inflammatory responses and compromised autonomic regulation. This review underscores the need for prospective, long-term studies to elucidate the relationships between hypertension and cognitive disorders and to evaluate the potential therapeutic benefits of antihypertensive treatments.

## 1. Introduction

Hypertension is a prevalent and persistent public health issue affecting more than one billion individuals worldwide [1], including more than 75% of persons older than 65 years [2]. Risk factors include age, obesity, diabetes, hyperlipidemia, family history, smoking, alcohol consumption, and physical inactivity [1]. Beyond its well-established cardiovascular implications such as stroke, myocardial infarction, and premature death, hypertension has been identified as a potentially modifiable risk factor for neurocognitive disorders in emerging studies [2,3,4].

The Diagnostic and Statistical Manual of Mental Disorders, Fifth Edition, Text Revision (DSM-5-TR) categorizes neurocognitive disorders into delirium and neurocognitive disorders, the latter further divided into major and mild neurocognitive disorders. These are then classified into thirteen etiological subtypes, including Alzheimer’s disease, vascular dementia, Lewy body dementia, and frontotemporal dementia [3,5]—the primary focuses of this literature review. Notably, while the DSM-5 updated the term “dementia” to “major neurocognitive disorder”, to reduce stigma and encompass a broader range of cognitive impairments, “dementia” is still widely used in the medical literature. For clarity, this review, when referring to undefined or general cognitive impairment, will refer to both as “major neurocognitive disorder”, a leading cause of disability and death worldwide, affecting nearly 55 million individuals [6].

Major neurocognitive disorder is a multifactorial condition influenced by a combination of genetic, environmental, and lifestyle factors. A family history of major neurocognitive disorder indicates potentially inherited susceptibility. Among the significant genetic risk factors is the APOE4 allele, which is linked to Alzheimer’s disease [6]. Additionally, there is a genetic correlation between low-density lipoprotein cholesterol levels and Lewy body dementia [7]. Furthermore, an individual’s personal medical history—including comorbidities such as hypertension, hypotension, cardiovascular disease, diabetes, chronic kidney disease, and type 2 diabetes [3,8,9]—contributes significantly to the overall risk of cognitive impairment. Understanding these risk factors provides a comprehensive foundation for considering the role of blood pressure in neurocognitive health.

The pathophysiological mechanisms linking hypertension to neurocognitive disorders are complex and bidirectional, often exploring the question of whether hypertension is a causative factor versus consequence of cognitive decline, both, or neither. The answer varies depending on the type of cognitive disorder as well as findings between observational and random controlled trials. Generally, chronic hypertension can lead to structural and functional changes in cerebral vasculature, including reduced cerebral blood flow and blood–brain barrier disruption [8]. Hypertension is often preceded by arterial stiffening, triggering adaptive remodeling responses throughout cerebral vasculature [10]. Vascular changes, inflammation, neurovascular dysfunction, and blood–brain barrier disruption threaten the health of cortical tissue and subcortical white matter, causing hypoperfusion and the disruption of metabolite clearance that contribute to network dysfunction, neuronal loss, and brain atrophy [10,11]. Conversely, that cognitive decline exacerbates cardiovascular risks through heightened inflammatory responses and compromised autonomic regulation [6]. Emerging research in this area has led to hypertension being targeted as a treatable condition that could delay cognitive decline, yet there is significantly conflicting data regarding antihypertensive treatment effects. This review will focus on the relationship between hypertension and cognitive disorders, elaborate on pathophysiology, and assess potential therapeutic approaches to mitigate these risks.

## 2. Delirium

Delirium is commonly observed in hospitalized older adults, and is defined by a disturbance in attention and awareness that develops over a short period of time, represents a change from baseline, and tends to fluctuate in severity throughout the day [12]. Delirium can be triggered by a variety of circumstances, including infection, medication, and surgery. The diverse phenotypes and underlying factors contributing to delirium pose significant challenges for identification, management, and research. Often occurring in the presence of pre-existing conditions such as prior cognitive impairment or comorbidities, delirium is associated with increased inflammation and heightened susceptibility to cerebral insults, including hypoperfusion and hypertension during hospitalization [13,14,15], as well as higher morbidity and mortality [16].

Management of delirium often includes the use of antipsychotic medications such as haloperidol to address symptoms like agitation and hallucinations. However, the efficacy of haloperidol remains controversial. A 2019 systematic review encompassing 16 randomized controlled trials and observational studies found no significant difference in outcomes compared to placebos [17], suggesting that there may be other mechanisms at play to target for delirium management.

One systematic review concluded that hypertension [18] was a risk factor for delirium, while another systematic review [13] identified other cardiovascular disorders as risk factors. Cohort studies have also identified hypertension as a significant risk factor for delirium. For example, one study, after adjusting for age and sex, examined delirium risk factors in 260 patients who were divided into medical and surgical subgroups; hypertension was a salient common risk factor [12]. Another study of 173 patients greater than 60 years of age reported that hypertension was an independent predisposing factor for delirium after cardiac surgery, with a strong odds ratio of 2.73 (95% CI: 1.16–6.40) [16]. Similarly, it was found in a cohort of 98 patients greater than 60 years of age that the incidence of postoperative delirium in the hypertension group was 41% compared with 12% in the non-hypertension group. To further ascertain blood pressure relation, the incidence of postoperative delirium in the irregular-medication group was 62% compared with 25% in the regular-medication group [15]. Generally, it is well supported that hypertension is a risk factor for delirium.

It should follow that antihypertensives mitigate risk or potentially treat delirium, as seen in a cohort of 5641 propensity score-matched inpatients greater than 65 years of age with trauma prescribed antihypertensive medication (angiotensin-converting enzyme inhibitors (ACEIs) and angiotensin receptor blockers (ARBs), calcium channel blockers (CCBs), beta blockers, and thiazides) 28 days before the injury [19]. The in-hospital incidence of delirium in the antihypertensive treatment group compared to nontreatment was lower, with a relative ratio of 0.55 (95% CI: 0.37–0.82). Moreover, ARBs were associated with the lowest incidence of delirium with the lowest odds ratio of 0.58 [19]. In a large-scale study involving 25.5 million individuals, which tracked delirium rates over a two-year period among patients prescribed antihypertensive medications, the findings ranked the incidence of delirium from lowest to highest as follows: ACEI/ARBs, CCBs, and beta blockers [20]. ACEI/ARBs have significant potential to reduce cognitive decline, potentially linked to the “angiotensin hypothesis” that plays a notable role in major neurocognitive disorder pathophysiology, which will be discussed further below. 

However, in a study that evaluated the risk between delirium prevalence and being prescribed ACEI and ARB in 4791 patients admitted to intensive care units (ICUs), there was no difference found in delirium rates among patients with no exposure to ACEI/ARB (12.6%) or exposure to ACEI (14.4%), ARB (11.8%), or both (15.4%) in six months prior to the ICU admission [14]. While ACEIs and ARBs stabilize the blood–brain barrier by inhibiting inflammatory responses that may contribute to delirium, the severe health conditions of ICU patients may have surpassed the protective effects of these medications [19].

Prospective studies on the effects of antihypertensive treatment on delirium are limited, likely due to the unpredictable and transient nature of delirium, which complicates the design of long-term controlled studies. Furthermore, hypertension is only one of many potential risk factors contributing to the heterogeneous nature of delirium [13,16,17]. Thus, while managing hypertension may theoretically reduce some risk, it is unlikely to fully resolve delirium on its own.

## 3. Alzheimer’s Disease

Alzheimer’s disease accounts for about 75% of all major neurocognitive disorder cases, characterized by the progressive deterioration of basic cognitive (episodic memory, linguistic, spatial, orienting) and executive functions (inhibitory abilities and visuospatial functioning) [21]. In Alzheimer’s disease, hypertension is associated with an increased number of neuritic amyloid-beta plaques in the neocortex and hippocampus as well as neurofibrillary tangles seen in autopsy studies [9]. Compromised vascular integrity leads to cerebral amyloid angiopathy and impaired beta-amyloid plaque clearance from the brain, which are hallmark findings of Alzheimer’s disease [6,22,23]. It is well established in research that mid-life hypertension from 45 to 64 years is consistently linked to later-life major neurocognitive disorder risk, including Alzheimer’s disease and vascular dementia, while late-life hypertension’s impact varies [6]. A post mortem analysis of Alzheimer’s disease brains found a significantly reduced spread of tau tangles and amyloid plaques in those who were regularly using antihypertensive treatment [24].

In a comprehensive review of the pathophysiology between hypertension and Alzheimer’s disease, it was concluded that hypertension may not innately increase one’s risk of Alzheimer’s disease, but likely exacerbates the pathogenesis [8]. However, in a two-sample Mendelian randomization analysis with a mediation study, it was found that stroke played a critical mediating role in the “causal” relationship between hypertension and Alzheimer’s disease, accounting for 55% of hypertension’s effect [25]. Other studies like The Morbidity and Mortality After Stroke, Eprosartan Compared With Nitrendipine for Secondary Prevention (MOSES), and Prevention Regimen for Effectively Avoiding Second Strokes (PROFESS) also assessed the pharmacological secondary prevention of stroke and found no differences in preventing cognitive decline [22].

A review of five meta-analyses and 52 primary studies found systolic blood pressure to be associated with an increased risk of Alzheimer’s disease by 11%, while no association was found for diastolic blood pressure [21]. In a cross-sectional cohort study of 67 patients over 60 years old with hypertension who reported mild cognitive impairment or subjective cognitive concerns, Pittsburgh compound B seen on positron emission tomography scans (PiB-PET), a marker of beta-amyloid retention, was found to be associated with worse episodic memory [26]. In contrast, white matter hyperintensities (WMHs), a marker of subcortical ischemic injury, did not show a significant association with performance in any cognitive domain [26]. This suggests that Alzheimer disease pathology may be driven by pathology other than hypertensive changes. These findings underscore the potential value of Alzheimer’s disease biomarker testing—such as PET amyloid imaging or a cerebrospinal fluid analysis—as a screening measure for patients with persistent, unexplained cognitive impairment or hypertension. However, a few studies have not demonstrated a clear link between hypertension and Alzheimer’s disease imaging biomarkers [26], especially in mid-life and early adulthood—the very periods during which studies suggest that hypertension significantly elevates the risk of Alzheimer’s disease [11,22]. Thus, while hypertension is a notable health concern, it is relatively weak as a risk factor for Alzheimer’s disease compared to other underlying conditions and factors that more directly contribute to major neurocognitive disorder.

To date, there are no widely accessible treatments that significantly change the course of Alzheimer’s disease, which is why primary prevention is critical [9]. In a comprehensive review of hypertension and the risk of major neurocognitive disorders, it was found that observational studies support the potential benefit of antihypertensives preventing cognitive decline seen in Alzheimer’s disease, but inconclusive evidence from randomized control trials and systematic reviews suggests otherwise [22]. In a cohort study of Medicare beneficiaries with hypertension, starting an antihypertensive medication regimen that stimulates, rather than inhibits, type 2 and 4 angiotensin II receptors was linked to a 16% lower risk of developing Alzheimer’s disease and related dementias over a follow-up period of approximately 7 years [27]. In a secondary longitudinal analysis from the Ginkgo Evaluation of Memory Study, involving 2248 older adults over a median of 6.1 years, participants with normal cognition who used antihypertensive medications showed varying degrees of reduced risk for developing Alzheimer’s disease. ARBs were the most effective (hazard ratio: 0.31), followed by ACEIs, diuretics, beta blockers, and CCBs (HR: 0.62) [28]. In participants with mild cognitive impairment, only diuretic use was linked to a decreased risk of Alzheimer’s disease [28]. In a meta-analysis that examined six prospective community-based studies of 31,090 adults over 55 years, with follow-ups ranging from 7 to 22 years, it was found that in hypertensive individuals, antihypertensive medication use was associated with a significantly reduced risk of developing major neurocognitive disorder (by 12%) and Alzheimer’s disease (by 16%) [29]. This particular study also assessed combination regimens; no specific antihypertensive medication class was found to be more effective than others in reducing major neurocognitive disorder risk. In participants with normal blood pressure, antihypertensive medication use did not significantly affect major neurocognitive disorder risk [29].

Generally, CCBs show the most potential as they readily cross the blood–brain barrier due to high lipophilicity; this same property could be why they are also associated with higher risk of delirium development [6,20]. ACEI and ARB have also been shown to slow the decline in memory function in individuals with mild to moderate Alzheimer’s disease, likely due to their anti-inflammatory effects [14] and involvement in the renin–angiotensin system.

## 4. Vascular Dementia

Vascular dementia and hypertension have a clearer association, where reduced blood flow to neurons results in cerebral atrophy [30]. The second most common form of major neurocognitive disorder, vascular dementia, often coexists with Alzheimer’s disease. It is important to note that vascular involvement in Alzheimer’s disease is very common, and that both diseases share symptomatology. It is unclear if vascular dementia and Alzheimer’s disease are additive or synergistic or represent a spectrum of the same cognitive disorder [24], which may lead to diagnostic confounding. Neuropsychological studies have shown that, unlike Alzheimer’s disease patients, those with vascular dementia tend to perform better on memory tests but worse on executive function tests, suggesting that executive dysfunction may be a potential diagnostic marker of vascular dementia [3]. Besides clinical markers, studies that have assessed WMH, a biological marker of subcortical ischemic injury, show decreases with antihypertensive and lipid-lowering treatment [30].

Hypertension in mid- (45 to 64 years) and late-life (65–74 years) has been found to be associated with an increased risk of vascular dementia [6]. In a study with 11,114 initial presentations of vascular dementia over a median follow-up of 7 years, it was found that blood pressure was positively associated with risk of vascular dementia, with a possible plateau at 120 mmHg, irrespective of a prior transient ischemic attack or stroke [31]. In a systematic assessment of antihypertensive treatment to prevent major neurocognitive disorder subtypes including vascular dementia, it was found that beta blockers and vasodilator antihypertensives showed lower risk of major neurocognitive disorder compared to other drug classes like diuretics [32]. Despite the fact that diuretics also increase angiotensin-II, these data do not adhere to the angiotensin hypothesis, so the mechanism may be more likely due to reduced vascular burden. Of interest, studies have found a U-shaped phenomenon between systolic blood pressure and probable Alzheimer’s disease and probable vascular dementia, suggesting that extremes of blood pressure contribute to higher cognitive decline risk [33].

## 5. Lewy Body Dementia

Lewy body dementia includes dementia with Lewy bodies and Parkinson’s disease dementia, defined as if the cognitive or motor symptoms arise first, respectively [6]. Characterized by fluctuating cognition, parkinsonism, visual hallucinations, and rapid eye movement sleep disorder, Lewy body dementia has relatively weaker understanding compared to other major neurocognitive disorder subtypes [7].

A retrospective cohort study of 7544 control participants, 1324 Alzheimer’s disease participants, and 562 Lewy body dementia participants found that hypertension was a common risk factor [34]. While the pathophysiologic relationship between hypertension and Lewy body dementia is less understood, the incidence is well established. In a study of 20 participants with Lewy body dementia who underwent imaging, data showed that cerebral microbleeds were present in 30%, and 100% of this group reported a history of hypertension [35]. In a population-based case–control study of 148,170 US Medicare participants diagnosed with Lewy body dementia, data showed significantly reduced Lewy body dementia risk with antihypertensives, cholesterol-lowering agents, and anti-diabetics [7].

Yet, studies on hypertension association with Lewy body dementia are sparse. It is better known that Lewy body dementia is associated with orthostatic hypotension through autonomic dysfunction, possibly through alpha-synuclein modulation. Hypotension is also a risk factor for dementia, as mentioned with the U-shaped phenomenon with Alzheimer’s disease and vascular dementia. For Lewy body dementia, hypotension is associated with a 26% relative increase in risk [9], explained through poor brain perfusion leading to subcortical infarction and ischemic demyelination.

## 6. Frontotemporal Dementia

Frontotemporal dementia is classified into behavioral and language variants that result from neuronal loss in the frontal and/or temporal lobes. Given its lower incidence and prevalence compared to other major neurocognitive disorder subtypes, there is lesser research on its relationship with hypertension [6].

A database review found that patients with cerebrovascular-disease behavioral-variant frontotemporal dementia were almost 10 years older at the onset of cognitive decline (71.6 vs. 62.5 years) and time of death compared to non-cerebrovascular behavioral-variant frontotemporal dementia. Additionally, the cerebrovascular group were more likely to have hypertension (75.8% vs. 45.7%) and history of stroke (21.2% vs. 6.1%), suggesting a strong association with vascular risk factors [36].

Some studies report similar hypertension prevalence in frontotemporal dementia to other neurocognitive disorders, while others show lower rates compared to Alzheimer’s [37,38]. While there are few studies that suggest a relationship between frontotemporal dementia and hypertension, a prospective study of 100 participants with frontotemporal dementia and 200 controls matched by age and sex concluded smoking, being overweight [6], and especially type 2 diabetes [39] as stronger risk factors for frontotemporal dementia.

In the same cross-sectional study assessing 100 frontotemporal dementia cases against 200 controls, no association in hypertension (65% vs. 67.3%) was found, or for dyslipidemia, obesity, and hypothyroidism [39]. The study found a significant association between frontotemporal dementia and diabetes, proposing that neurodegeneration in frontotemporal dementia occurs in brain regions with high densities of insulin receptors that are sensitive to changes in central nervous system insulin signaling [39]. Additionally, it is reasonable to hypothesize that hypertension may accelerate this process through the formation of advanced glycation end products, which contribute to oxidative stress and vascular damage, potentially exacerbating neurodegenerative processes in vulnerable brain regions.

## 7. Undefined Cognitive Impairment

The majority of research investigating the relationship between hypertension and cognitive impairment does not define cognitive impairment clearly [12,13]. Additionally, inconsistencies in defining mid-life and late-life, varying hypertension thresholds, and difficulties in clinically distinguishing neurocognitive disorders—whether through a diagnosis or autopsy—further cloud the understanding of this relationship. The lack of specificity poses challenges for accurately assessing and comparing research findings, especially across the thirteen etiological neurocognitive disorder subtypes outlined in the DSM-5. For instance, while mid-life (45–64 years) hypertension increases risk of both Alzheimer’s disease and vascular dementia, this strength of association is less evident in Lewy body dementia and frontotemporal dementia [6], highlighting unique pathophysiological bases. This section will focus on addressing these inconsistencies by exploring how undefined cognitive impairment is approached in the literature, highlighting the need for more precise definitions to improve the validity and reliability of future research.

Less is understood regarding the correlation between high blood pressure and cognitive deficits in specific domains like episodic memory, semantic verbal fluency, fluid reasoning, and numerical ability [40]. In a study with 337 participants with a history of a clinical high-blood-pressure diagnosis of a mean age of 48.78 ± 17.06 years compared with 26,707 healthy controls of a mean age of 45.30 ± 15.92, it was observed that participants with high blood pressure demonstrated impaired performance in all these cognitive domains [40]. While the study was cross-sectional, longitudinal studies could illuminate how elevated blood pressure affects different cognitive domains over time. For example, understanding whether working memory, not assessed in the study, declines more rapidly than numerical ability could guide healthcare providers in designing interventions, akin to promoting crossword puzzles for cognitive preservation. A notable limitation of the study is its focus on patients with a history of a clinical high-blood-pressure diagnosis, lacking details on medication and treatment regimens [40]. Additional investigations could explore whether patients on specific antihypertensive therapies showed varied cognitive outcomes, strengthening this association and suggesting protective therapeutic effects. In a study of 1385 participants diagnosed with mild cognitive impairment, it was shown that a higher degree of high blood pressure (systolic blood pressure ≥ 140 mmHg or diastolic blood pressure ≥ 90 mmHg) was associated with a faster decline in certain cognitive function domains. Notably, high systolic blood pressure was prevalent in 63% of participants compared to high diastolic readings in 20% of participants [11,41].

Several meta-analyses support the angiotensin hypothesis, which posits that several antihypertensive medication classes lower major neurocognitive disorder risk by stimulating the angiotensin-II receptors’ type (ATR) 2 and 4, involved in cerebral ischemia and memory function [42], having overall an anti-cholinergic effect. ARBs block ATR1, increasing ATR2 and ATR4 stimulation, and upregulate angiotensin-II production [23,27]. Beta blockers and CCBs decrease renin and subsequently, angiotensin-II. It is consistent in the literature that ARBs, beta blockers, and CCBs attenuate cognitive decline, although it is unclear whether aggressive treatment during what age or stage of hypertension would be the most effective, e.g., in mid-life [10]. ACEIs inhibit angiotensin-II production, which aligns with most data [43] that ACEIs have a relatively lesser effect in reducing major neurocognitive disorder incidence [42]. In a recent observational cohort study, it was found that ARBs, beta blockers, CCBs, and diuretics were associated with a 14–35% lower major neurocognitive disorder risk compared to ACEIs, which aligns with previous observational studies and meta-analyses agreeing that ARBs have the best effect on the lowest risk of major neurocognitive disorder compared to other antihypertensive classes [42].

A review of 12 randomized control trials, totaling 30,412 participants, assessed whether pharmacological treatment of hypertension can prevent cognitive impairment, with trials ranging from 2 to 5 years, and ultimately concluded low certainty [44]. The variability in these studies [6,9], as well as general differences in blood pressure targets and cognitive outcome definitions outlined in a review (seen across the Systolic Hypertension in Europe (Syst-Eur) trial, the Hypertension in the Very Elderly Trial (HYVET), and the Action to Control Cardiovascular Risk in Diabetes (ACCORD) trial) [2], leaves the effect of intensive blood pressure control on cognitive impairment, especially in different age groups, uncertain. Few studies have had a follow-up of longer than 4 years, and some observational studies have even identified an association between low blood pressure and higher risk of cognitive impairment [2].

Other studies report antihypertensive treatment reducing risk of cognitive impairment [45,46], with the notable Honolulu Asia Aging Study following elderly men who were hypertensive since mid-life over > 12 years. It was concluded that the longer the duration of antihypertensive medication use, the significantly lower the risk for major neurocognitive disorder [47]. Indeed, the data also suggested that long-term antihypertensive treatment could slow the rate of cognitive decline in nondemented subjects [47], possibly offering protective mechanisms for overall brain health and mitigating age-related changes or preventing toxic accumulation, whether beta-amyloid or advanced glycation end products. A notable limitation of the study is that it focused on Japanese American men only, and while the findings are impressive, they cannot be generalized to the broader population.

The SBP Intervention Trial-Memory and Cognition in Decreased Hypertension (SPRINT MIND) is one of the more significant antihypertensive studies designed to attenuate cognitive impairment, assessing intensive SBP lowering (goal: <120 mm Hg) versus standard treatment (goal: <140 mm Hg) on the prevention of mild cognitive impairment and major neurocognitive disorder among 9361 participants [2,4,11,33], reporting a significant association of blood pressure control with decreased risk of cognitive impairment.

## 8. Conclusions

The reviewed literature underscores a complex and multifaceted relationship between hypertension and neurocognitive disorders, including delirium, Alzheimer’s disease, vascular dementia, Lewy body dementia, and frontotemporal dementia. Overall, evidence suggests that hypertension is a significant risk factor for various forms of cognitive impairment, with differing implications depending on the specific neurocognitive disorder and the age of onset.

The relationship between hypertension and cognitive disorders is complex and multifaceted, involving a combination of vascular, inflammatory, and neurodegenerative processes. Understanding these mechanisms is essential for developing effective interventions to prevent or delay cognitive decline in hypertensive individuals. Hypertension is challenging to isolate, as it often is associated with aging, which naturally is associated with general impairment of cerebral blood flow, microvascular pressure, and cellular stress resistance, as well as other comorbidities such as obesity, diabetes, and hyperlipidemia—all risk factors for dementia [8]. Indeed, it is important to acknowledge that primary and secondary hypertension have different mechanisms. Few studies explore these relationships in detail, while several exclude secondary hypertension causes from their analyses [43]. This raises the question of whether hypertension itself directly contributes to cognitive decline or if underlying secondary conditions play a more significant role [21], highlighting the need for more targeted research in future studies. Furthermore, lifestyle factors such as obesity, smoking, stress, and other social determinants of health (explored in previously mentioned studies with Medicare beneficiary cohorts) further complicate the interplay between hypertension and major neurocognitive disorder. Beyond inconsistencies mentioned in “Undefined cognitive impairment”, several studies highlight potential confounders such as age, comorbid conditions, and medication adherence, which could affect the outcomes related to hypertension and major neurocognitive disorder.

Continued research into the pathophysiology of hypertension and its impact on the brain will pave the way for novel therapeutic strategies aimed at improving cognitive health and quality of life for older adults. Current recommendations for blood pressure control in relation to cognitive health, particularly in elderly patients, remain unclear. The SPRINT MIND trial provided valuable insights by directly investigating the impact of intensive blood pressure control on cognitive outcomes. However, aggressive blood pressure management in older adults may introduce risks such as syncope, orthostatic hypotension, and falls [2]. Given these considerations, a personalized approach that carefully weighs the cognitive benefits of tighter blood pressure control against the potential risks of overtreatment is advised. Future studies with larger sample sizes and longitudinal designs are needed to identify the optimal systolic and diastolic blood pressure ranges that best protect cognitive function. While ARBs and CCBs show promise due to their anti-inflammatory properties and ability to cross the blood–brain barrier, further research will help refine treatment regimens to better meet individual patient needs. A summary of antihypertensive treatment on major neurocognitive disorders discussed is provided in Table 1.

While there have been global advancements in detecting hypertension, treatment and control rates vary significantly. Factors such as the unequal access to medications, lack of universal healthcare, and poor implementation of targeted public health measures may contribute to these low control rates of 23% for women and 18% for men, with even lower rates in low- and middle-income countries where hypertension is increasingly prevalent [9]. The economic impact of major neurocognitive disorder is estimated at USD 600 billion per year worldwide, highlighting its importance as a public health priority [8].

## Figures and Tables

**Table 1:** Overview of Major Neurocognitive Disease Subtype, Association with Hypertension, and Effect of Antihypertensive Treatment Trials.

Major Neurocognitive Disorder Subtype	Association with Hypertension	Antihypertensive Treatment Trials
Delirium	Hypertension increases risk of delirium through increased inflammation and heightened susceptibility to cerebral insults.	Delirium incidence is lower with antihypertensive treatment; ACEI/ARB > CCBs. Limited prospective data.
Alzheimer’s disease	Hypertension accelerates Alzheimer’s disease pathology, with increased risk in mid-life (45–64 years) and variable risk in late life.	Conflicting data from randomized control trials and systemic reviews; ACEI/ARBs > CCBs.
Vascular dementia	Hypertension increases risk in mid- (45–64 years) and late life (65–74 years).	Beta blockers > renin–angiotensin system agents.
Lewy body dementia	Hypertension is a known risk factor, although pathophysiology is not well understood.	Limited pharmacologic trial data available. One case control study, n = 148,170 participants with Lewy body dementia with decreased risk from antihypertensive, lipid-lowering, and anti-diabetic agents [7].
Frontotemporal dementia	Associated with a variety of cardiovascular risk factors like obesity, diabetes, and dyslipidemia. Limited studies on hypertension association.	Limited pharmacologic trial data available.
Undefined cognitive impairment *	Hypertension is associated with various forms of cognitive impairment.	Most meta-analyses support the angiotensin hypothesis—angiotensin-II stimulators attenuate cognitive decline.

Table 1 provides an overview of the dementia subtypes discussed in this review, highlighting their associations with hypertension, along with antihypertensive treatment trial result summaries. * Limited validity due to variations in hypertension definitions and cognitive disorder diagnostic criteria.

## Data Availability

No new data were created or analyzed in this study. Data sharing is not applicable to this article.
